# Interaction of the p53 DNA-Binding Domain with Its N-Terminal Extension Modulates the Stability of the p53 Tetramer

**DOI:** 10.1016/j.jmb.2011.03.047

**Published:** 2011-06-10

**Authors:** Eviatar Natan, Cetin Baloglu, Kevin Pagel, Stefan M.V. Freund, Nina Morgner, Carol V. Robinson, Alan R. Fersht, Andreas C. Joerger

**Affiliations:** 1 MRC Laboratory of Molecular Biology, Hills Road, Cambridge CB2 0QH, UK; 2 Physical and Theoretical Chemistry Laboratory, Department of Chemistry, University of Oxford, South Parks Road, Oxford OX1 3QZ, UK

**Keywords:** DBD, DNA-binding domain, nESI-MS, nanoflow electrospray ionization mass spectrometry, PDB, Protein Data Bank, TAD, transactivation domain, HSQC, heteronuclear single quantum coherence, tumor suppressor, protein structure, cation–π interaction, oligomerization, electrospray mass spectrometry

## Abstract

The tetrameric tumor suppressor p53 plays a pivotal role in the control of the cell cycle and provides a paradigm for an emerging class of oligomeric, multidomain proteins with structured and intrinsically disordered regions. Many of its biophysical and functional properties have been extrapolated from truncated variants, yet the exact structural and functional role of certain segments of the protein is unclear. We found from NMR and X-ray crystallography that the DNA-binding domain (DBD) of human p53, usually defined as residues 94–292, extends beyond these domain boundaries. Trp91, in the hinge region between the disordered proline-rich N-terminal domain and the DBD, folds back onto the latter and has a cation–π interaction with Arg174. These additional interactions increase the melting temperature of the DBD by up to 2 °C and inhibit aggregation of the p53 tetramer. They also modulate the dissociation of the p53 tetramer. The absence of the Trp91/Arg174 packing presumably allows nonnative DBD–DBD interactions that both nucleate aggregation and stabilize the interface. These data have important implications for studies of multidomain proteins in general, highlighting the fact that weak ordered–disordered domain interactions can modulate the properties of proteins of complex structure.

## Graphical Abstract

**Figure:**
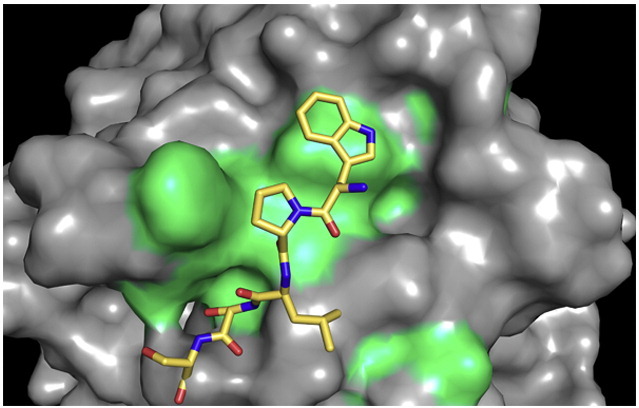

## Introduction

The tumor suppressor p53 plays an essential role in the control of the cell cycle and the protection of the genome.^1,2^ It functions primarily as a transcription factor by binding to specific DNA response elements as a homotetramer and integrates a multitude of cellular signals via protein–protein interactions to initiate the appropriate cellular response. This functional diversity and adaptability is provided through a complex domain structure, which comprises intrinsically unfolded N- and C-terminal extensions and linker regions that flank the structured DNA-binding domain (DBD) and tetramerization domain.^3–5^ The p53 tetramers consist of dimers of dimers, with a *K*_d_ in the low nanomolar range.^6–11^ We have previously shown by mass spectrometry that a p53 construct comprising the DBD and tetramerization domain but not the termini, p53(94–360), exchanges its subunits relatively slowly on a cellular timescale,^12^ with important implications for its biological activity, cellular localization and the dominant-negative effect of mutant over wild-type p53.

**Fig. 1:**
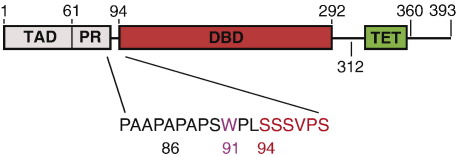
Domain structure of p53. The 393-aa p53 protein has an intrinsically disordered N-terminal region comprising the TAD and a proline-rich region (PR). The structured DBD (red) and tetramerization domain (TET) are connected via a flexible linker. Like the N terminus, the C-terminal region is also disordered. See the text for further details. The hinge region between the canonical DBD region and the PR, containing the structurally important Trp91, is shown in detail. In addition, domain boundaries of p53 constructs used are highlighted.

A combination of NMR, small-angle X-ray scattering and electron microscopy revealed that the full-length p53 protein has an open, cross-shaped structure with loosely coupled DBD dimers in its DNA-free form, whereas the structure rigidifies upon DNA binding and becomes more compact.^13,14^ The transactivation domain (TAD) at the N terminus (residues 1–60) is intrinsically disordered and can be divided into two subdomains (TAD1 and TAD2) responsible for binding to negative regulators, transcriptional coactivators and components of the transcription machinery.^15–18^ It is followed by a proline-rich region, which favors a polyproline II helix structure and serves as a rigid linker to project the TAD away from the DBD.^19^ A short, poorly characterized hinge region connects this domain to the canonical region of the DBD, extending from residues 94–292 (Fig. 1). Crucially, the factors contributing to thermodynamic and kinetic stability of the tetramer, as well as the dynamics of domain–domain interplay, particularly the role of linker regions, are only poorly characterized at the level of the full-length protein. Previous studies on N-terminally truncated p53 constructs have generally been done on sequences beginning at residue 94.^20–22^

In about 50% of human cancers, the p53 protein is inactivated by mutation. Most p53 cancer mutations are located in the DBD.^23,24^ This domain is thermodynamically and kinetically unstable. Many oncogenic mutations lower the stability of the DBD even further as a result of structural perturbations, causing it to rapidly unfold at body temperature while retaining native conformation at subphysiological temperature.^24–28^ Such temperature-sensitive cancer mutants are potential drug targets and can, in principle, be reactivated by small-molecule binders that preferentially bind to the native state, thus preventing unfolding.^29,30^ It has been suggested that aggregation of structurally destabilized p53 cancer mutants plays an important role in tumor induction or progression.^31^ Exposure of an aggregation-prone sequence in the DBD upon unfolding not only results in a mere loss of function but causes an aggregation-dependent dominant-negative effect of mutant over wild-type p53 in heterozygous cells, in addition to impairment of wild-type activity via formation of mixed tetramers. Moreover, this aggregation propensity accounts for mutant p53 oncogenic gain of function via co-aggregation with the paralogs p63 and p73, which blocks potential p53 salvage pathways.^31^ A recent study on p53(94–360) variants suggests that the unfolding of mutant DBD is accelerated in tetrameric p53 as a result of an increase in the local concentration of DBDs,^21^ although this phenomenon has not been investigated using full-length proteins yet. A key question when studying multidomain proteins is whether the behavior of isolated domains or combinations thereof reflects that of the full-length protein.

Here, we used NMR to map intramolecular interactions in various p53 domain constructs. Subsequent X-ray crystallography revealed that Trp91, outside the usually defined canonical region of the DBD, folds back onto the latter and forms a cation–π stacking interaction with the guanidinium group of Arg174 in the DBD. Most notably, the absence of this N-terminal extension changes the kinetics of tetramer assembly and aggregation properties, presumably as a result of nonnative DBD interactions, thus providing insights into the dynamic nature of the full-length protein. These data highlight the importance of studying the contribution of the individual subdomains for a detailed description of the full-length protein.

## Results

### NMR studies reveal interaction between the N terminus and the DBD of p53

**Fig. 2:**
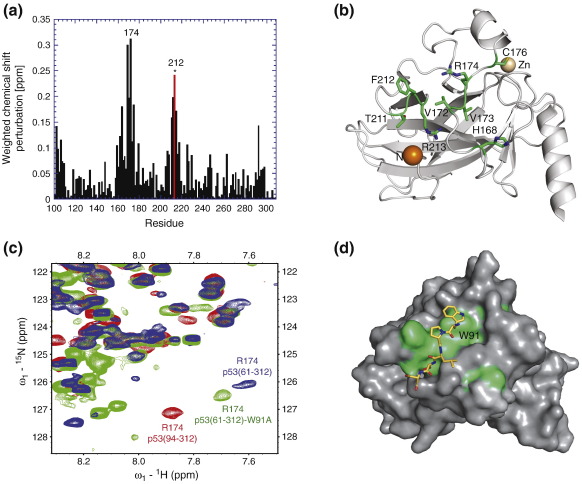
Mapping of p53 DBD interactions. (a) Chemical shift perturbations in p53(86–312) compared to p53(94–312). Phe212 could not be traced in p53(86–312) because of the overlapped position of this resonance (highlighted in red and marked with an asterisk). (b) Ribbon diagram of the structure of T-p53(94–312) (chain A, PDB ID 1UOL).^32^ The N-terminal residue Ser96 is highlighted with an orange sphere. Residues showing the largest perturbations when comparing the spectra of p53(94–312) and p53(1–312)/p53(86–312) are shown as green stick models. (c) Overlay of the HSQC spectra of p53(94–312) (red), p53(61–312) (blue) and p53(61–312)-W91A (green); the chemical shift position of Arg174 is labeled. (d) Surface representation of p53(94–312) shown in the same orientation and with the same color code as in (b). The yellow stick model shows the conformation of the N-terminal extension, starting with Trp91, in the crystal structure of p53(89–293).

To map possible interactions of the p53 N terminus with the DBD, we conducted NMR measurements using constructs comprising the DBD (residues 94–312) with either full N terminus [p53(1–312), residues 1–312] or truncated N terminus [p53(61–312), residues 61–312; p53(86–312), residues 86–312] as well as the full-length p53 (see Fig. 1). ^15^N,^1^H heteronuclear single quantum coherence (HSQC) spectra of p53(1–312) and the isolated DBD p53(94–312) overlaid well for most DBD residues apart from significant chemical shift perturbations for residues His168, Val172, Arg174, Cys176, Thr211, Phe212 and Arg213. The largest chemical shift perturbation was observed for Arg174, indicative of a significant interaction of this residue with the N terminus (Fig. 2). All perturbed residues cluster to a surface patch on the DBD, which might act as a docking site for hydrophobic/aromatic residues (Fig. 2b). Further analysis of chemical shift perturbations observed for DBD constructs with truncated N terminus as well as constructs with mutated residues in key hydrophobic motifs in the N terminus (TAD1/TAD2) allowed us to define N-terminal residues involved in the interaction with the DBD. p53(1–312) variants with mutations of evolutionary conserved hydrophobic residues in TAD1 (L22Q/W23S) and TAD2 (W53Q/F54S) exhibited very similar spectra as the p53(1–312) variant. Apart from some local chemical shift perturbations at the mutation sites, no significant changes were observed for DBD residues, indicating that the highly conserved hydrophobic motifs within TAD1 and TAD2 are not involved in an interaction with this domain. This result was confirmed by the spectra of p53(61–312) and p53(86–312) with partly truncated N termini, which were almost identical with those of p53(1–312) containing the full N terminus. The region of the N terminus interacting with the DBD could, therefore, be pinpointed to residues 86–93, the hinge region between the structured DBD and the proline-rich region. This region contains a tryptophan (Trp91), which most likely packs against the surface patch around Arg174/Phe212 on the DBD. In agreement with this hypothesis, chemical shift positions for key residues (e.g., Arg174) in the core domain surface patch in the W91A mutant of p53(61–312) were in between the values observed for p53(61–312) and p53(94–312), suggesting that Trp91 is a key interacting residue (Fig. 2c). Arg174 in full-length p53 had the same chemical shift as in p53(1–312), p53(61–312) and p53(86–312), indicating that the intramolecular interaction of the hinge region with the patch around Arg174 is retained in tetrameric full-length p53.

### Trp91 forms a cation–π interaction with Arg174

**Fig. 3:**
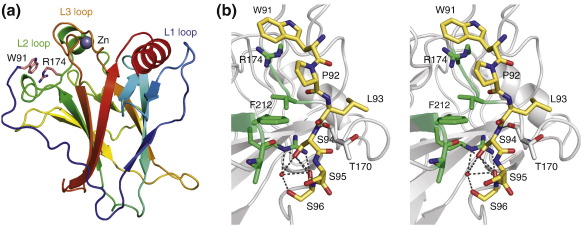
Crystal structure of the extended p53 DBD. (a) Ribbon diagram shown with a rainbow color gradient from blue at the N terminus to red at the C terminus. The side chains of Trp91 and Arg174, which form a cation–π interaction, are shown as stick models. (b) Stereo view of the N-terminal region. Residues 91–96 are shown as yellow stick models. Selected side chains in the L2 loop region that interact with the N terminus are shown as gray and green stick models. The hydrogen-bond network involving Ser94 is highlighted with broken lines.

In order to elucidate the exact nature of the interaction detected by NMR, we solved the crystal structure of a DBD construct comprising residues 89–293 at 1.68 Å resolution (Table 1). The crystals belonged to space group *P*2_1_ with two molecules in the asymmetric unit. The overall structure of the β-sandwich and the DNA-binding region, including the zinc coordination sphere, is essentially the same as reported for the DBD variants with a shorter N terminus (Fig. 3a). The C^α^ atoms of residues 96–290 in both chains superimpose with the equivalent atoms in the four chains in Protein Data Bank (PDB) ID 2OCJ^34^ with a root-mean-square deviation (RMSD) of 0.3–0.6 Å, which is in the range of the RMSD when superimposing the chains within the asymmetric unit. In both subunits of our structure with elongated N-terminal tail, the N-terminal residues up to Trp91 fold back onto the DBD, in excellent agreement with the analysis of local chemical shift perturbations by NMR in solution (Fig. 2d). A sharp turn within residues 94–96 facilitates this folding back of the N terminus against the L2 loop region of the DBD. The turn is stabilized via a hydrogen-bond network involving the hydroxyl group of Ser94, which is within hydrogen-bond distance of the main-chain oxygen of Thr211, the main-chain nitrogen and oxygen of Ser96 and a highly coordinated structural water molecule (Fig. 3b). A further hydrogen bond links the amide nitrogen of Ser94 with the carbonyl group of Thr170. The C^β^ atom of Pro92 packs against the aromatic ring of Phe212. Key in positioning the extreme N terminus is a cation–π interaction of the indole ring of Trp91 with the guanidinium group of Arg174, which packs in an approximately coplanar manner, the preferred geometry in protein structures.^35^ The average vertical distance between the guanidinium and the indole plane is about 3.6 Å, and the interplane angles are 5° and 7° in chains A and B, respectively. The orientation of the N-terminal residues is essentially the same in both subunits. Residues 91–99 of both chains superimpose with an RMSD of 0.31 Å for all equivalent atoms. For the two preceding residues, Pro89 and Ser90, no electron density was observed, indicating that they are highly flexible and do not significantly interact with the folded region of the DBD but point away from the structured domain.

### Thermodynamic stability of the extended DBD

The thermodynamic stability of full-length p53 is dictated by the stability of its DBD.^36^ We used differential scanning fluorimetry to investigate whether the segment around Trp91 contributes to the thermodynamic stability of the DBD (Table 2). The melting temperature *T*_m_ of p53(89–293) was increased by 0.6 °C (*T*_m_ = 46.4 °C) relative to that of p53(94–312), which lacks the N-terminal extension but contains parts of the linker region between the DBD and the tetramerization domain. The difference in stability was even more pronounced in tetrameric p53 variants that differed solely in their N-terminal extension, as p53(89–360) showed a 2 °C increase in *T*_m_ compared to p53(94–360). These data show that the p53 DBD is stabilized through additional interactions mediated by its N-terminal extension.

### Aggregation properties of p53 domain variants

**Fig. 4:**
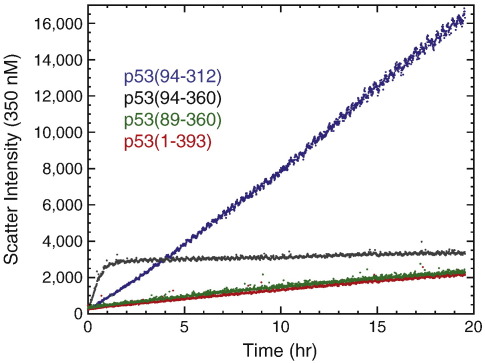
Aggregation of different p53 variants at 37 °C. Light-scattering curves at 350 nm show that p53(94–360) is much more prone to aggregation than the isolated DBD (residues 94–312), p53(89–360) and the full-length protein at a protein concentration of 3 μM (monomers).

We used light scattering at 350 nm to monitor the aggregation of different p53 domain constructs at 37 °C over a time course of 16–20 h (Fig. 4). The scatter intensity for p53(94–360) increased rapidly and reached saturation after about 2 h . In contrast, the scatter intensities for p53(89–360) and full-length p53 increased much more slowly and did not reach saturation within the time of the experiment. These data show that p53(94–360) is much more prone to aggregation than the other constructs, having by far the shortest half-life. The scattering curves of p53(89–360) and the full-length protein were almost identical, suggesting that the short segment from residues 89–94 plays an important role in preventing accelerated aggregation of the full-length p53 tetramer.

The isolated DBD, p53(94–312), at the same concentrations, showed a different pattern of light scattering—the time course was linear in the same time range, showing that it was still in the growth phase and yet reached much higher intensities despite not having proceeded to completion (Fig. 4). The higher intensity shows that the DBD forms much larger aggregates, which have inherently higher scattering effects than small aggregates, than did p53(94–360).

### Intramolecular and intermolecular interactions modulate the subunit exchange of the p53 tetramer

**Fig. 5:**
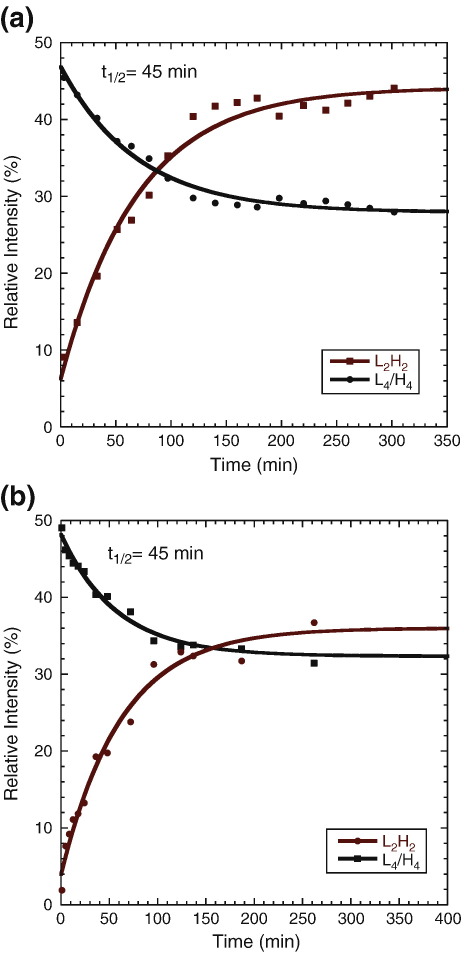
Kinetics of subunit exchange of tetrameric p53. Equal amounts of unlabeled (“L”, ^12^C–^14^N  ) and uniformly labeled (“H”, ^13^C–^15^N  ) p53 protein were mixed followed by nESI-MS analysis of the species present at various time points after mixing. The kinetic modeling of subunit exchange for (a) full-length p53 and (b) p53(89–360) at 20 °C shows that they have the same exchange rate.

Nanoflow electrospray ionization mass spectrometry (nESI-MS) studies showed that the p53 variant comprising the DBD and the tetramerization domain, p53(94–360), exchanges its subunits relatively slowly, with a half-life of 160 min for the exchange of dimeric subunits at 20 °C.^12^ We have now used the same protocol of mixing equal amounts of unlabeled protein (^12^C–^14^N) and uniformly labeled protein (^13^C–^15^N), followed by nESI-MS analysis at various time points after mixing to measure the subunit exchange for p53(89–360) and the full-length p53 protein at 20 °C. As with p53(94–360), only the exchange of dimers but not monomers was observed within the experimental timescale. Full-length p53 exchanged its dimeric subunits with a half-life of 45 min (Fig. 5a), which is 3.55 times faster than the subunit exchange in p53(94–360). Hence, the presence of the terminal regions quenches some of the intersubunit contacts observed in p53(94–360). Most interestingly, the half-life for exchange of p53(89–360) subunits was the same as that of the full-length protein (Fig. 5b). These data suggest that, by folding back onto the DBD, the region around Trp91 buries a surface patch on the DBD that is responsible for nonnative intersubunit contacts in p53(94–360) that slow down subunit exchange.

## Discussion

The folding and assembly of individual domains of p53 have been studied in much detail, yet relatively little is known about how these processes are modulated in the full-length protein. By combining stability data, measurements on the subunit exchange by nESI-MS and structural studies to map domain–domain interactions in the full-length protein, we were able to provide new insights into the interplay between different domains in full-length p53 and its effect on the stability of the p53 tetramer. Surprisingly, the subunit exchange in p53(94–360) was significantly slower than in the full-length protein, suggesting that there are additional, nonnative domain–domain interactions that are either not present or attenuated in the full-length protein. Addition of only a few residues preceding the canonical core domain region (including Trp91) was sufficient to prevent this phenomenon, highlighting the intricate oligomerization properties of human p53. Another level of complexity is added by the recent discoveries that interactions with accessory proteins of the S100 family^38^ or 14-3-3 isoforms^39,40^ further modulate tetramer formation by interacting with regions within and outside the p53 tetramerization domain. Hence, the stability of the p53 tetramer not only is regulated by competing intrasubunit and intersubunit contacts but also critically depends on the biological context and the phosphorylation state of p53.

A study of p53(94–360) variants with cancer-associated mutations reported that tetramer formation drastically increases the propensity of oncogenic p53 mutants to misfold compared to monomeric DBD. The authors concluded that tetramerization establishes an efficient trap for p53 misfolding by increasing the local DBD concentration.^21^ Interestingly, we observed a similar proximity effect when measuring the aggregation of p53(94–360) at body temperature, which proceeded much faster than in the case of the isolated DBD. Yet, importantly, this effect was quenched in tetrameric constructs with an extended N terminus, as p53(89–360) and the full-length protein were much less prone to aggregation. Most likely, exposure of a surface patch in p53(94–360), either fully or transiently buried in the full-length protein, results in artificially high aggregation tendencies because of enhanced nonnative DBD interactions upon tetramer formation. Our structural and biophysical data suggest that folding back of the N-terminal DBD extension around Trp91 onto the DBD structure plays a key role in preventing these interactions and, hence, accelerated aggregation of the p53 tetramer. As such, the increased aggregation tendency and the slower subunit exchange of p53(94–360) may have the same structural basis. The importance of regulating p53 aggregation properties has been highlighted by a recent study showing that aggregation of structurally destabilized cancer mutants is responsible for oncogenic gain of function through co-aggregation with the p53 family members p63 and p73.^31^ These data further emphasize the special structural constraints on complex, oligomeric signaling proteins such as p53 to prevent malignant transformations. They require sufficient, tightly controlled half-lives and structural safeguards against accelerated unfolding and aggregation. However, at the same time, they have to retain enough structural plasticity for both cooperative DNA binding and interactions with their numerous partner proteins in the cell cycle.

**Fig. 6:**
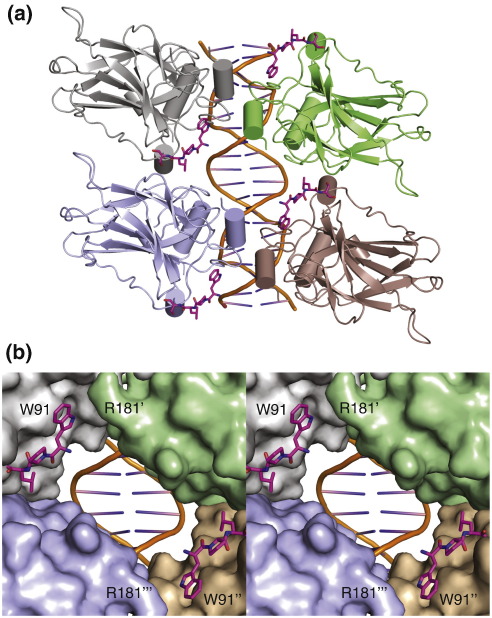
Location of the additional N-terminal DBD residues with respect to the interface regions in the p53–DNA complex. (a) Chain A of the DBD structure with extended N terminus was superimposed onto each chain of the structure of four p53 DBDs assembled onto a full DNA response element without spacer between the two half-sites (PDB ID 3KZ8).^43^ DNA-bound molecules are shown as cartoon representations. Residues 91–95 of the unbound structure are shown as a magenta stick model, suggesting that folding back of the N-terminal region via Trp91 is compatible with productive DNA binding by contributing to the translational DBD–DBD interface and projecting the proline-rich region away from the protein–DNA interface. (b) Close-up stereo view of the central cavity in (a) highlighting the proximity of Trp91 to Arg181 from an adjacent DBD at the interface between two DBDs bound to a DNA half-site and the space available for the N-terminal extension in the full-length protein. DNA-bound DBDs are shown as surface representations, and the N-terminal extension is shown as a magenta stick model. Orientation and color code are the same as in (a).

Our structural data argue in favor of revising the current domain boundaries of the p53 DBD, which, historically, has been defined to start somewhere between residues 94 and 102 based on proteolytic digests and crystallographic studies.^22,41^ The short segment around Trp91, although not essential for the overall structure of the DBD, contributes to the thermodynamic stability of the DBD by folding back against a nonnative surface patch on the canonical DBD region. Key to this interaction is the formation of an energetically favorable cation–π interaction between Trp91 and Arg174 (Fig. 3). Trp91 is strictly conserved in mammalian p53, suggesting a similar structural role. Its counterparts on the surface patch, Arg174 and Phe212, are also either strictly or highly conserved in mammalian p53. Two recent papers report the structure of four p53 DBDs (in both cases without Trp91) assembled on a continuous p53 response element without spacer between the two half-sites, typical of the majority of p53 response elements.^42,43^ In one of these structures, which has two additional N-terminal residues, Leu93 is part of the translational interface between two DBD dimers bound to a DNA half-site,^42^ which is in direct vicinity of the docking site for the N-terminal tail, and superimposes very well with the position of the Leu side chain in our structure. This dimer–dimer interface depends on the nature of the response element (i.e., the length of the spacer between half-sites), and the pattern of sequence conservation follows approximately that of Trp91. Superimposing our structure with an extended N terminus onto both DNA complexes suggests that packing of Trp91 onto Arg174 is compatible with productive DNA binding, as it leaves a possible trajectory for the N terminus without perturbation of the p53–DNA complex (Fig. 6). As such, Trp91 may play an important role in positioning the proline-rich region. Another interesting aspect of this model is the fact that the indole nitrogen of Trp91 is within approximately 4.5 Å of the guanidinium group of Arg181 from an adjacent subunit, which is part of the symmetrical DBD–DBD interface (within a half-site), raising the possibility that Trp91 contributes to this interface via a water-mediated contact. DNA-binding studies *in vitro* have shown that binding of the full-length protein to different p53 response element sequences exactly parallels the DNA-binding affinities measured for p53(94–360), implying that the N-terminal extension, including Trp91, has no significant effect on sequence-specific DBD–DNA interaction.^44^

In summary, our data show that the stability of the full-length p53 tetramer is determined through a dynamic interplay of intermolecular and intramolecular interactions. The short segment from Trp91 to Ser94 modulates the stability of the p53 tetramer by capping the structured region of the DBD and, thus, forms an integral part of the DBD. The altered molecular surface of the extended DBD structure has, for example, implications for p53 drug discovery and should be taken into account when designing generic small-molecule binders for rescuing the function of temperature-sensitive p53 cancer mutants.^3^ Importantly, the data highlight that studies on truncated variants do not fully reflect the complexity of the full-length protein, a general problem when studying multidomain proteins, which are highly abundant in human signaling networks. The energetics of the additional DBD–DBD interdomain interactions are relatively small—a decrease in dissociation rate constant of a factor of 3.55 is equivalent to 0.75 kcal/mol of activation energy. Similarly, the free energy of denaturation of p53(89–360) is only 0.75 kcal/mol higher than that of p53(94–360), calculated from the increase in *T*_m_ of 1.9 °C and data from our previous work.^36^ Thus, small changes in energy can have significant effects on such properties as subunit dissociation and protein aggregation when these events are on a timescale of several minutes upwards.

## Materials and Methods

### Protein expression and purification

Unless stated otherwise, the p53 constructs used contained four stabilizing mutations (M133L/V203A/N239Y/N268D) in the DBD.^32,37^ Expression and purification of these stabilized p53 variants followed published protocols.^13^ Briefly, the p53 variants were produced in *Escherichia coli* BL21(DE3) as a fusion protein with N-terminal 6×His/lipoamyl domain/tobacco etch virus protease cleavage site. The proteins were purified using standard His-tag purification protocols, followed by tobacco etch virus protease digestion, heparin affinity chromatography and a final gel-filtration step. The genes of wild-type p53(89–293) and p53(94–312) were expressed in *E. coli* C41(DE3) without an N-terminal tag using a pET-24a(+) vector, as described previously for several DBD mutants,^45,46^ and the proteins were purified by cation-exchange chromatography on a Sepharose SP column (GE Healthcare), affinity chromatography on HiTrap Heparin (GE Healthcare) and gel filtration on a Superdex 75 column (GE Healthcare) as the final purification step. Expression of ^15^N- and ^13^C-labeled p53 was carried out in M9 minimal medium supplemented with vitamins, minerals and 1 g/L ^15^NH_4_Cl and ^13^C d-glucose.

### NMR

All two-dimensional HSQC spectra were acquired on Bruker Avance 600- and  800-MHz spectrometers equipped with triple-resonance TXI cryoprobes at protein concentrations of 50–450 μM in 25 mM sodium phosphate buffer, 150 mM NaCl, 5 mM DTT and 5% D_2_O (v/v). Standard triple-resonance experiments were used to confirm the resonance assignments in the p53(1–312) construct. All data were processed in Topspin (Bruker, Karlsruhe), referenced against the internal water signal and analyzed in Sparky (Goddard and Kneller, SPARKY 3, University of California, San Francisco). The weighted ^15^N,^1^H chemical shift perturbations (in parts per million) were calculated according to |Δδ(^1^H)| + |Δδ(^15^N)|/5 based on the observed individual chemical shift differences Δδ^1^H and Δδ^15^N (in parts per million) utilizing in-house perl scripts with drift correction (T. J. Rutherford).

### X-ray crystallography

Crystals of the DBD with extended N terminus (residues 89–293) were grown by sitting-drop vapor diffusion at 17 °C. One microliter  of protein solution [5.4 mg/mL in 25 mM sodium phosphate (pH 7.2), 220 mM NaCl and 5 mM DTT] and 1 μL reservoir solution [100 mM 2-[bis(2-hydroxyethyl)amino]-2-(hydroxymethyl)propane-1,3-diol (pH 5.5), 23% polyethylene glycol 3350 and 200 mM ammonium sulfate] were mixed above a reservoir of 85 μL. Crystals grew within a few days. They were soaked in mother liquor supplemented with 10% glycerol and flash frozen in liquid nitrogen. An X-ray data set was collected at the Diamond Light Source (beamline I04) and was processed with MOSFLM^47^ and SCALA.^48^ The structure was solved by molecular replacement with Phaser^49^ using chain A of PDB ID 1UOL^32^ as a search model. Subsequent structure refinement was performed using PHENIX,^50^ and manual model building, with Coot.^51^ Data collection and refinement statistics are summarized in Table 1. Structural figures were prepared with PyMOL‡.

### Subunit exchange and mass spectrometry

In a typical subunit exchange reaction, equal amounts of [^12^C–^14^N]p53 and [^13^C–^15^N]p53 ([p53]total = 10 μM, in tetramers) in either 250 mM (full-length p53) or 500 mM [p53(89–360)] ammonium acetate, pH 6.9, were mixed and incubated at 20 °C. At different time points after mixing, samples were taken, and nanoflow electrospray ionization mass spectra were recorded on either a Q ToF II or a Synapt HDMS system (Waters Corp., Milford, MA) optimized for the transmission of noncovalent complexes.^52^ The following instrument parameters were used: (i) Q ToF II: capillary voltage, 1.5 kV; sample cone, 100 V; transfer extractor cone, 40 V; accelerating voltage into the collision cell, 10 V; ion transfer stage pressure, 3 × 10^− 3^ mbar; and ToF analyzer pressure, 1.34 × 10^− 6^ mbar. (ii) Synapt HDMS: capillary voltage, 1.0–1.5 kV; sample cone, 50 V; cone gas, off; trap and transfer collision voltage, 50 V; ion transfer stage pressure, 4.40 mbar; trap pressure, 4.95 × 10^− 2^ mbar; ion mobility cell pressure, 4.34 × 10^− 1^ mbar; and ToF analyzer pressure, 1.17 × 10^− 6^ mbar. Calibration, data processing, spectra simulation and kinetic modeling were performed as described previously.^12^

### Differential scanning fluorimetry

Thermal unfolding of p53 was followed by monitoring the binding of the dye SYPRO Orange (diluted 5000-fold; Invitrogen) using a Rotor-Gene 6000 real-time PCR thermocycler (Qiagen) with a scan rate of 270 °C/h in 25 mM sodium phosphate (pH 7.2), 150 mM NaCl and 5 mM DTT. The sample size was 20 μL, and the protein concentration was 10 μM (monomers). Experiments were carried out in quadruplicate. Data analysis was performed with the software supplied by the manufacturer.

### Light-scattering measurements

Aggregation of different p53 variants at 37 °C was measured by light scattering in a FluoroMax-3 spectrofluorometer equipped with a thermostatted cell holder at an excitation and emission wavelength of 350 nm. Spectra were recorded over a time course of 16–20 h . The slit widths for excitation and emission were 0.5 and 1.0 nm, respectively. The protein concentration was 3 μM (monomers) in 25 mM sodium phosphate (pH 7.2), 150 mM NaCl and 5 mM DTT.

### Accession numbers

The atomic coordinates and structure factor amplitudes for the extended p53 DBD have been deposited in the PDB§ (PDB ID 2XWR).

## Figures and Tables

**Table 1:** X-ray data collection and refinement statistics

*Data collection*	
Space group	*P*2_1_
Cell, *a*, *b*, *c* (Å)	51.50, 68.45, 58.89
Cell, α, β, γ (°)	90.00, 99.46, 90.00
Molecules per asymmetric unit	2
Resolution (Å)^a^	44.3–1.68 (1.77–1.68)
Unique reflections	46,147
Completeness (%)^a^	99.5 (99.4)
Multiplicity^a^	3.4 (3.3)
*R*_merge_ (%)^a^^,^^b^	5.7 (22.8)
〈*I*/σ_*I*_〉^a^	13.5 (4.5)
Wilson *B* value (Å^2^)	17.9

*Refinement*	
Number of atoms	
Protein^c^	3181
Water	510
Zinc	2
*R*_cryst_ (%)^d^	17.3
*R*_free_ (%)^d^	20.9
RMSD bonds (Å)	0.006
RMSD angles (°)	1.08
Mean *B* value (Å^2^)	20.8
Ramachandran favored (%)^e^	99.0
Ramachandran outliers (%)^e^	0.0

a Values in parentheses are for the highest-resolution shell.

b *R*_merge_ = ∑(*I*_*h,i*_ − 〈*I*_*h*_〉)/∑*I_h,i_*.

c Number includes alternative conformations.

d *R*_cryst_ and *R*_free_ = ∑||*F*_obs_| − |*F*_calc_||/∑|*F*_obs_|, where *R*_free_ was calculated over 5% of the amplitudes chosen at random and not used in the refinement.

e Determined using MolProbity.^33^

**Table 2:** Melting temperature of p53 variants

p53 variant^a^	Apparent *T*_m_ (°C)
p53(94–312), wild-type DBD	45.8 ± 0.2
p53(89–293), wild-type DBD	46.4 ± 0.1
p53(94–360)	50.6 ± 0.1
p53(89–360)	52.5 ± 0.1

a The tetrameric p53 variants contained the stabilizing mutations M133L/V203A/N239Y/N268D (Joerger *et al*.^32^ and Nikolova *et al*.^37^).

## Footnotes

‡ www.pymol.org

§ www.pdb.org
